# The Use of MALDI‐TOF MS for Microbial Identification of Discolored Vacuum‐Packaged Beef

**DOI:** 10.1111/1750-3841.70591

**Published:** 2025-10-10

**Authors:** Alejandro Poveda, Johannes Krell, Volker Heinz, Nino Terjung, Igor Tomasevic, Monika Gibis

**Affiliations:** ^1^ DIL German Institute of Food Technologies Quakenbrück Germany; ^2^ Department of Food Material Science, Institute of Food Science and Biotechnology University of Hohenheim Stuttgart Germany

## Abstract

Vacuum‐packaging technologies have been developed over recent decades to extend the shelf‐life of beef and to provide consumers with standardized, high‐quality products. However, muscles stored under vacuum can still undergo discoloration, likely when animals are exposed to adverse pre‐mortem weather conditions (high air humidity and/or large temperature fluctuations). Even if microbial activity is assumed to play a role in discoloration processes, relationships between bacterial populations and beef discoloration remain largely unexplored. This manuscript aims to identify potential microbiological germs related to discoloration in vacuum‐packaged beef. To assess the microbiological basis of beef discoloration, total viable and anaerobic counts were quantified and bacterial identification using MALDI‐TOF MS was performed in both discolored (*n* = 12) and control (*n* = 12) meat groups. The samples were collected at approximately 5–8 days post‐mortem from the storage facility of a German slaughterhouse. Unexpectedly, the pH values in the control samples were consistently higher than those in the discolored samples, however a significant color difference was found (*p *< 0.05). Discolored muscles appeared to have higher lightness (L*) values, but had lower redness (a*) and overall color intensity (*h*_a/b_) compared to the control samples, which can also be associated to higher superficial metmyoglobin formation. Principal component analysis (PCA) and network plot were performed using eight different variables of the data matrix. Both statistical tools confirmed that there were differences between discolored and control groups when measured at both test points. It seemed that the microbial activity in both groups of samples could not entirely explain the discoloration processes happening during beef aging in vacuum‐packages.

## 1 Introduction

Intricate unitary operations during muscle‐to‐meat conversion can critically influence final product quality (Chambers et al. 2001; Braden 2013). Following slaughtering, the shift from aerobic to anaerobic metabolism leads to pH decline due to lactic acid accumulation, which consequently triggers different physico‐chemical pathways that affect meat quality, including color (Poveda‐Arteaga et al. 2023). Color plays a central role in the purchasing decisions of food products, due to an evolutionary instinct developed throughout human history, in which red tonalities are related to nutritious and tasty food (Ruedt et al. 2023). In beef, red muscles are preferred over purple, brownish, or greenish. Meat pieces which do not meet these expectations are usually rejected, resulting in both economic losses and environmental waste (Tomasevic et al. 2019; R. C. Mancini and Hunt 2005; Ruedt et al. 2023). Traditionally, color is assessed visually or instrumentally, using tools like colorimeters and spectrophotometers, with high correlation between the two methods (R. R. Mancini et al. 2022; Ruedt et al. 2023). Instrumental methods focus on measuring three color coordinates, L* (lightness), a* (redness), and b* (yellowness) to detect possible oxidative changes in the surface of meat (Hernández et al. 2019). Hue (*h*_a/b_) and chroma (C*) are derived from the primary color coordinates and provide additional insights on how color is processed by the human eye (Hernández et al. 2019). *h_a/b_* refers to the overall color in relationship to wavelength, while C* represents the saturation of the red color (Gagaoua et al. 2018; Maciel et al. 2021).

Beef color is influenced by several intrinsic and extrinsic factors that interact between each other during pre‐ and post‐mortem operations (Poveda‐Arteaga et al. 2023; Faustman and Cassens 1990). The complexity of these associations result in inconsistent variability in beef carcasses, which makes it very complicated to predict and/or standardize meat quality traits (Clinquart et al. 2022). As an effective strategy to reduce these differences, the meat industry commonly employs aging to homogenize and enhance tenderness and palatability (Nair et al. 2019; Vitale et al. 2014). This procedure is commonly categorized into dry‐ and wet‐aging (Suman et al. 2014; F. W. Bischof et al. 2022). Dry‐aging was the standard procedure before the development of vacuum‐packaging techniques and consists on the direct exposure of beef carcasses to the environment at approximately 2°C and 75% relative humidity, which favors the growth of *Pseudomonas* (Gill 1983; McSharry et al. 2020; G. Bischof 2023). It is also known that a lack of vacuum in this form of packaging could also contribute to discoloration if a lower oxygen partial pressure was present (King et al. 2023; Denzer et al. 2025). On the other hand, wet‐aging consists on the storage of beef sub‐primals under vacuum packaging for around 7 and 21 days (Suman et al. 2014) at temperatures between −1°C to 2°C (Terjung et al. 2021) and promotes the proliferation of facultative‐ and strictly‐anaerobic microorganisms in meat, such as lactic acid bacteria (LAB), Enterobacteriaceae and *Clostridium* species (Dainty and Mackey 1992; Albrecht and Correa 2016). Vacuum‐packaging is considered as a safer microbiological practice due to the protective barrier of the plastic film, which inhibits the growth of aerobic microorganisms, although it may also change the sensory profile of meat (Terjung et al. 2021; Hernández‐Macedo et al. 2011).

High water activity and nutrient availability of meat provide an ideal medium for microbial growth, particularly of psychrotropic microorganisms, leading to meat spoilage, production of off‐odors and ‐flavors, lipid oxidation and discoloration (Zwirzitz et al. 2020; Domínguez et al. 2019; Farmer and Farrell 2018; Shao et al. 2022). Hence, microbiological identification of meat throughout the cold chain can provide valuable conclusions into understanding potential quality defects that may affect product integrity over time. In this study, matrix‐assisted laser desorption/ionization time‐of‐flight mass spectrometry (MALDI‐TOF MS) was employed as a rapid and cost‐effective method for microbial identification in food systems (Pavlovic et al. 2013; Altakhis et al. 2021; Singhal et al. 2015). This technique has been successfully applied to poultry and lamb meat, providing a high genus‐level agreement with 16S rRNA sequencing (Altakhis et al. 2021; Höll et al. 2016).

This study aimed to identify the potential causes of meat discoloration, with a particular focus on microbial activity, by analyzing discolored and control samples of vacuum‐packaged beef collected under commercial conditions at two testing points: after packaging and before the end of shelf‐life. Previous research has primarily relied on experimental models, such as inoculating specific bacterial groups into color‐stable and color‐labile muscles to mimic discoloration (Colton et al. 2024, Colton et al. 2024). Others have worked on microbial identification in meat packaged under modified atmosphere packaging (MAP) conditions, either under refrigerated (Ercolini et al. 2006) or super‐chilled environments (X. Yang et al. 2020; J. Yang et al. 2024). To the best of our knowledge, this represents the first attempt to characterize the dynamics of microbial populations of fresh, discolored, vacuum‐packaged beef.

## 2 Materials and Methods

### 2.1 Sample Preparation

Animals were processed under standard commercial conditions in a German cattle specialized slaughterhouse (Böseler Goldschmaus, Low Saxony, Germany). Upon arrival at the abattoir, cattle from the same trucks were moved, distributed, and kept in separate pens in the lairage area to prevent mixing between unfamiliar individuals. The animals were then slaughtered following stunning by a captive bolt, suspended by a hind leg, and exsanguinated. Next, the carcasses were halved, classified by a trained inspector and stored in the cold chambers at approximately 2°C for around 96 h. Following deboning, the beef subprimals were vacuum‐packaged and wet‐aged until commercialization.

### 2.2 Sample Collection

**FIGURE 1:**
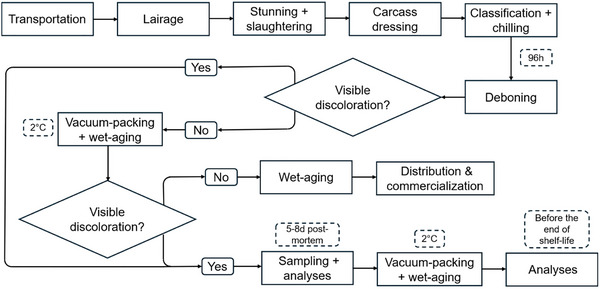
Flow‐diagram of the sampling and analysis procedure for discolored and control steaks.

During a pre‐trial phase, a total of 3 weeks was dedicated to visually inspecting discoloration issues at the end of the deboning line in the slaughterhouse. After this initial evaluation, it was concluded the probability to detect discoloration problems at this stage was negligible. However, discolored steaks could be identified following the processing line, when the meat was already vacuum‐packaged and stored for wet‐aging before distribution, as detailed in Figure 1.

Consequently, a total of 30 appointments were planned over a 4‐month span (from late March to late June) to identify and collect discolored (*n* = 12) and control (*n* = 12) samples from the wet‐aging storage facility of the aforementioned slaughterhouse. In each visit, around 30–50 packages were visually assessed. During this sampling period, the average daily air temperature was 14.5°C and the average daily air humidity was 69.6%, as detailed in Table 1.

**TABLE 1:** Weather conditions during the visit and sample collection days at the slaughterhouse from March through June.

	Number of samples	*T*_max_ air (°C)	*T*_min_ air (°C)	*T*_mean_ air (°C)	SD T air	RH air (%)
March	0	16	3	10.17	2.62	73.33
April	11	19	0	10.62	3.93	75.39
May	1	21	4	16.87	3.59	75.66
June	0	29	8	20.45	2.37	53.9

The samples collected during storage were categorized as discolored‐ and control‐after packaging. They consisted of parts of *M. gluteobiceps*, *M. tensor fasciae latae*, and *M. lateral vastus*, which had between 5 and 8 days post‐mortem and were analyzed for their qualitative and quantitative microbiological status, pH, and color. In addition, two samples of around 100 g each were separated from each steak and reserved for a repeated series of experiments before the steaks reached the end of shelf‐life. These samples were referred to as discolored‐ and control‐samples before expiration date. It was also seen that the discoloration in the affected steaks persisted throughout the entire storage period, up until the expiration point.

### 2.3 Quantitative Microbiological Analysis

A quantitative analysis was conducted for total viable counts (TVC) and anaerobic plate counts according to DIN ISO 4833‐2:2022 standards. For this purpose, the vacuum‐package of the discolored steaks was disrupted, and two samples of approximately 10 g were aseptically removed and identified as either: discolored‐after packaging or discolored‐before expiration date. The same procedure was applied for the control steaks to obtain the control‐after packaging and control‐before expiration date. The samples were immediately vacuum‐repackaged using a Thermoformer F5 packaging machine (Sealpac GmbH, Oldenburg, Germany). The packaging material was a mixture of polyamide and polypropylene that effectively restricted the exchange of oxygen, water, nitrogen, and CO_2_ between the interior and the environment (VF Verpackungen, Sulzberg, Germany). Discolored‐ and control‐after packaging samples were analyzed the next day after collection; while samples catalogued as discolored‐ and control‐before expiration date were stored at 2°C and only analyzed 1 day before the end of shelf‐life established for the slaughterhouse. Bacterial counts were expressed as log_10_ CFU/g.

### 2.4 Qualitative Microbiological Analysis

The individual identification for microbial populations were conducted using the MALDI‐TOF (MS) method, based on the DIN‐EN‐ISO 16140–4 standard, for specific microbial validation in food and feed matrixes (Bundesamt für Verbraucherschutz und Lebensmittelsicherheit 2021).

### 2.5 Color Measurement

Once the samples for the microbiological analysis were separated, the instrumental L*, a*, and b* values were measured, without allowing any blooming time. Briefly, color estimations were done using a portable ColorLite sph870 colorimeter (Katlenburg‐Lindau, Germany), with 45°/0°measuring geometry and 8 mm aperture size. The illuminant used was D65 with the aperture set to 10°. The instrument was previously calibrated using black and white ceramic tiles according to the manufacturer's specifications. Measurements were taken directly over the surface of the meat, with 15 readings every time, and the mean was used for data analysis. In addition, *h_ab_* and C* were calculated using Equations (1) and (2) (King et al. 2023) as follows:

(1) $$h_{\mathrm{ab}}=\arctan\left(\frac{b^{*}}{a^{*}}\right)$$

(2) $$C^{*}=\sqrt{a^{{*}^{2}}+b^{{*}^{2}}}$$

### 2.6 pH Measurement

The pH of the samples was measured by inserting the sensor of a portable Testo 205 pH meter directly into the meat (Lenzkirch, Germany). Previously, the glass sensor was calibrated with standardized buffer solutions at pH 4.00 and 7.00 at chilling temperature, which was the working temperature at the slaughterhouse. The pH measurement was performed at three different points inside the muscle and the mean was calculated.

### 2.7 Statistical Analysis

Descriptive statistics and a *t*‐test were carried out using SPSS software (version 23.0, IBM Corporation, Armonk, NY, USA) to find statistical differences between the variables in our study at a significance level of 0.01. In addition, principal component analysis (PCA) with covariance matrix and standardized scores, and a network plot using a 0.95 correlation coefficient were generated with Origin Pro (OriginLab Corporation, 01060 Northhampton, MA, USA).

## 3 Results and Discussion

Under commercial conditions, cattle slaughtering is an extremely precise and systematic practice with increasing complexity when larger volumes of animals need to be handled. Slaughterhouses must carefully adjust their operations based on several factors, including storage capacity, availability of raw materials, and the local working calendar. Meticulous planning is required to ensure the production of high‐quality and standardized meat, to the greatest extent possible.

The weekly activities of the abattoir, where the samples were collected, were organized in such a way that the carcasses of the animals slaughtered on Mondays, Tuesdays, and Wednesdays were chilled for approximately 48 h until being deboned and packaged. In contrast, the cattle carcasses slaughtered on Thursdays and Fridays were kept in the chilling chambers over the weekend, until they were further processed the following week. This implied that these carcasses were cooled for around 96 h before packaging. Curiously, the discolored samples collected for this manuscript came from animals slaughtered either on Thursdays or Fridays. However, finding discolored steaks in the rest of the slaughtering days could not be completely discarded (based on previous sampling, data not shown).

**FIGURE 2:**
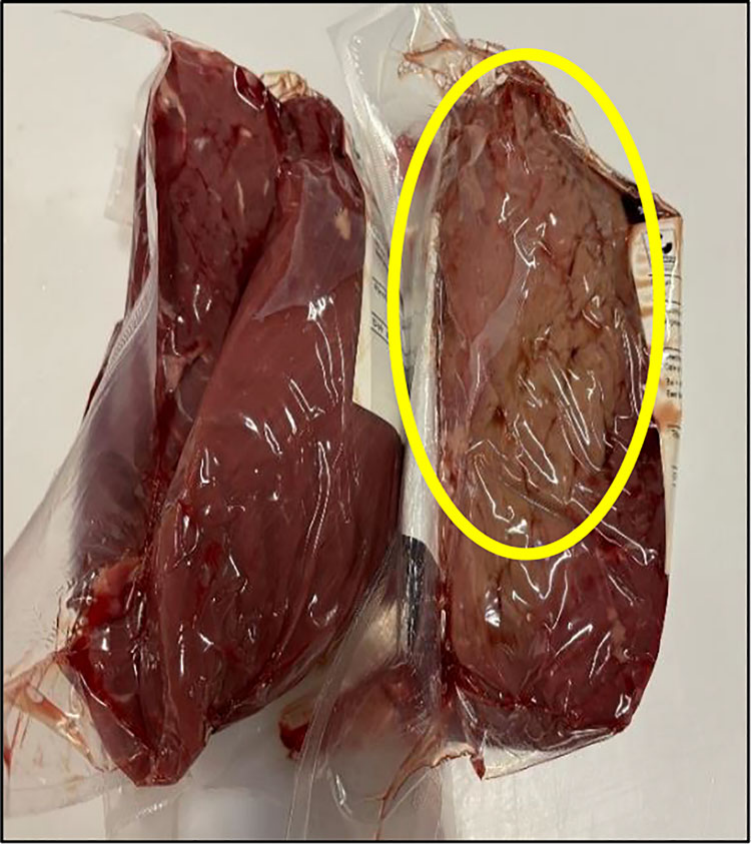
Visual differences between discolored‐ and control‐after packaging beef muscles (5–8 days post‐mortem).

Postmortem muscle tissue remains biochemically active, as several enzymatic and non‐enzymatic pathways that can affect meat color continue to function (Ramanathan, Hunt, et al. 2020). After slaughtering, as a result of a longer oxygen exposure, the myoglobin fractions present in the meat surface tended to stabilize the towards oxymyoglobin (King et al. 2023; Krell et al. 2024). As time post‐mortem continues, deoxymyoglobin formation from oxymyoglobin is favored, due to low‐oxygen partial pressures, that occur when oxygen is consumed, between other factors, by mitochondrial respiration or microbial growth (Wang et al. 2021; Denzer et al. 2024). The competition between mitochondria and myoglobin for the remaining oxygen has been described to play a central role in determining the final color of meat (Krell et al. 2024; King et al. 2023; Denzer et al. 2025). From here, it exists the possibility that metmyoglobin can be built beneath the surface of the muscles, between the oxymyoglobin and deoxymyoglobin layers, especially when critical oxygen partial pressure values, of around 1%–3% are reached (Krell et al. 2024; Ramanathan, Hunt, et al. 2020). It seemed that even after deboning (48–96 h post‐mortem), metmyoglobin was still not visible at this point, probably due to the action of inherently reducing enzymes present on fresh meat (King et al. 2023; A. E. Bekhit and Faustman 2005). However, a few days after vacuum‐packaging, metmyoglobin could have started to be built underneath the muscle surface, facilitated by oxygen diffusion that created a gradient that could have favored its accumulation (Ramanathan, Kiyimba, et al. 2020). As storage time increases, the efficiency of inherent metmyoglobin reduction systems in the meat decline, allowing this metmyoglobin layer to expand. Eventually, this metmyoglobin layer can migrate to the meat surface, resulting in visual discoloration (Denzer et al. 2024; King et al. 2023). Therefore, it is possible that some of those muscles could exhibit superficial discoloration problems that were optically identified from the control samples, as seen in Figure 2. A previous study has identified 1.3–1.6 log CFU/g higher microbiological counts in color labile muscles (*psoas major*), than in their color stable counterparts (*longissimus lumborum*) at the end of aerobic retail display (Colton et al. 2024). A similar experiment using the same muscles, explained an increased microbial growth in inoculated *psoas major* in comparison with *longissimus* (Colton et al. 2024). These findings reinforced the connection between meat discoloration and microbial growth kinetics, based in muscle‐specific differences. Microbial growth in combination with intrinsic muscle type differences seemed to rule the color behavior of meat (Denzer et al. 2024). Also higher oxygen consumption rates and less effective metmyoglobin reduction systems in *psoas major* muscles affected their color stability and produced increased metmyoglobin formation during aging and retail display, when compared to *longissimus lumborum* (Denzer et al. 2024). These differences in microbial load were, however, not seen in this study, probably because our samples had a similar muscular origin. Interestingly, discolored samples were found during 3 of the 30 visits (twice in April and once in May), which accounted for a 10% probability of finding any kind of discoloration issues during these months. It is important to mention that April and May were precisely the months with higher relative humidity and temperature standard deviations (Table 1). All discoloration incidents occurred in the meat from animals slaughtered either on Thursdays or Fridays, suggesting that these carcasses spent approximately 96 h in the chilling chambers before reaching the deboning line.

In our study, it was noticed that the likelihood of finding discoloration problems increased during cold, rainy days with large temperature fluctuations. It was previously postulated that under these stress conditions, increased shivering of the animals occurred, which resulted in lower glycogen reserves, and higher mitochondrial biogenesis. These issues were associated with defective color values in beef (Ramanathan, Kiyimba, et al. 2020). However, the reasons for this empirical finding are still unknown and more information about the pre‐mortem conditions needs to be collected to confirm this hypothesis. Apart from this, the establishment of effective and robust traceability systems is advantageous not only to identify potential food security hazards that might occur during meat processing, but they can also be a very strong tool to detect probable quality defects. In our case, traceability operations that could track back the data of the individual animals were only available until the beginning of the deboning line. After this point, the muscles from different animals were mixed during the following processes. This meant that the traceability was only feasible to back to the slaughtering and expiration dates, but it was no longer existing at an individual animal level. This limitation negatively affected the scope of the results, since the assessment of the influence of some of these pre‐ and post‐mortem factors were lost in the process.

**FIGURE 3:**
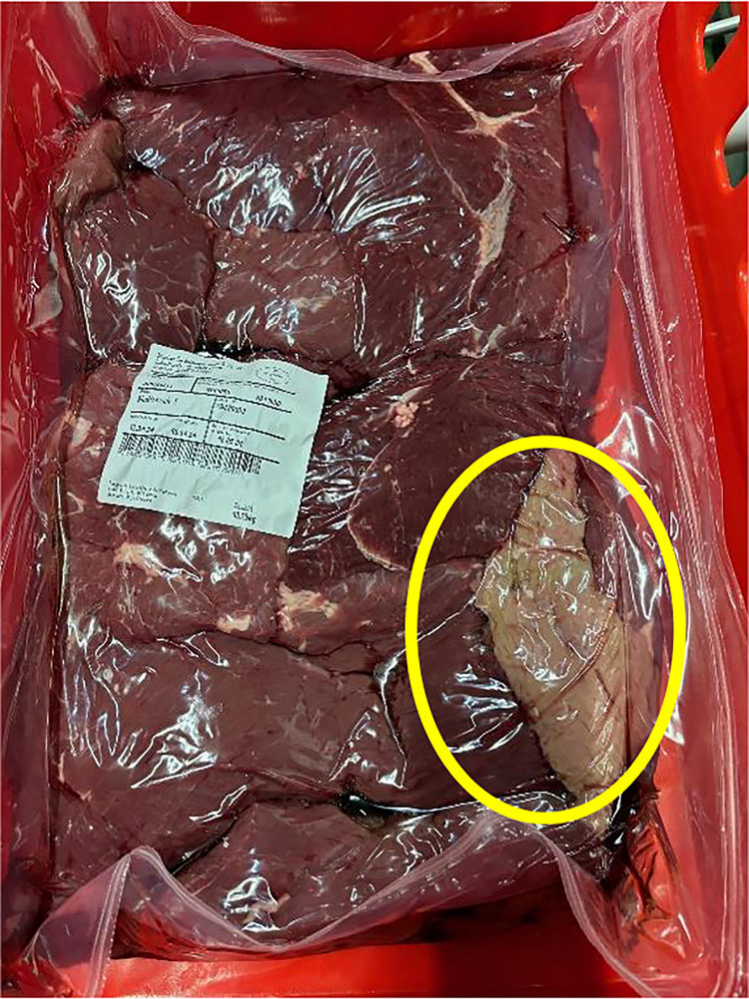
Sampling of discolored and control samples from a big package containing around six to eight muscles. It can be seen that not all muscles are affected by discoloration.

It is also worth mentioning that not all muscles from a single package were affected. Interestingly, only one or two cuts in the package displayed signs of discoloration, while the majority (typically 6–8 cuts) showed a standard purple color due to higher presence of deoxymyoglobin under vacuum‐packaging as shown in Figure 3. This means that even if adverse weather conditions could contribute to enhance discoloration problems in beef muscles, they were probably still dependent on individual animal intrinsic factors (Sullivan et al. 2022). It also can imply that discolored muscles did not interfere with the color of the rest of the meat pieces, which may explain that the reasons for this phenomenon are not entirely microbiological.

### 3.1 pH Values

Meat ultimate pH is one of the most critical parameters that affects the oxygen diffusion in post‐mortem animal muscle, thus defining meat color (Neethling et al. 2017; Poveda‐Arteaga et al. 2023; Ruedt et al. 2023). Any alterations in the pH of post‐mortem muscle can affect the structure and the redox status of myoglobin (Ramanathan, Hunt et al. 2020). If pH values fail to drop to values lower than 5.87 in post‐mortem beef muscle, then the condition known as dark, firm, dry (DFD) may occur. This meat quality defect is commonly related to stressful pre‐mortem conditions, which resulted in beef muscles with a darker appearance than normal, due to increased water capacity and more tightly packed fibers (Page et al. 2001; Ramanathan, Kiyimba, et al. 2020; English et al. 2016). In our experiment this quality defect could be virtually excluded, because none of the meat groups at any analysis point reached this pH threshold value.

From a microbiological point of view, bacteria that are typically responsible for meat spoilage tend to lower the pH values of the meat matrix (Lorenzo et al. 2018). Changes in the pH of vacuum‐packaged meat can also change the conditions in which bacteria grow, resulting in the production of different types of metabolites, depending on the substrates utilized (A. Bekhit et al. 2021; Hughes et al. 2014). The pH values of the discolored meat group in our experiment dropped by 0.3 points during wet‐aging time, probably due to a higher lactic acid accumulation (Table 2). A similar meat pH decline was previously documented from vacuum‐packaged beef, and it was assumed to be caused for the same reasons (Hanna et al. 1983; Hughes et al. 2014; Iulietto et al. 2015).

**TABLE 2:** Average quality traits of discolored (*n* = 12) and control (*n* = 12) beef samples collected during storage and measured right before the expiration date.

	After packaging^a^		Before expiration date^a^	
Parameter	Discolored meat (Da)	Control (Ca)	Discolored meat (Db)	Control (Cb)
pH	5.58 ± 0.5^b^	5.64 ± 0.8^a^	5.55 ± 0.8^b^	5.67 ± 0.1^a^
L*	35.9 ± 4.9^a^	21.0 ± 4.1^b^	34.9 ± 5.4^a^	27.6 ± 13.6^b^
a*	17.6 ± 2.1^b^	24.3 ± 5.1^a^	14.7 ± 2.6^b^	21.6 ± 7.8^a^
b*	18.7 ± 4.0^a^	14.7 ± 3.2^b^	12.9 ± 5.7^a^	12.3 ± 6.1^a^
*h_a/b_*	46.2 ± 5.19^a^	31.6 ± 6.7^b^	39.9 ± 8.9^a^	28.9 ± 9.7^b^
C*	25.8 ± 1.2^a^	28.6 ± 5.1^a^	19.8 ± 1.6^a^	25.1 ± 9.18^a^
TVC (log_10_ CFU/g)	3.30 ± 0.6^a^	3.05 ± 0.8^a^	6.24 ± 1.0^a^	5.92 ± 1.1^a^
Anaerobic counts (log_10_ CFU/g)	2.82 ± 0.8^a^	2.4 ± 1.1^a^	5.1 ± 1.0^a^	5.09 ± 1.6^a^

^a^ Different letters indicate significant differences between discolored samples and controls at the same time.

Interestingly, the mean pH value of the control samples increased by 0.3 points during wet‐aging. These results are akin to a previous investigation, which explained that the changes in muscle biochemistry probably affected the development of bacterial populations (Hughes et al. 2014). It has been explained that glucose is the primary metabolite to be exhausted by microorganisms in meat, followed by lactic acid (A. Bekhit et al. 2021). It is possible that due to the absence of carbohydrates available for microbial growth, bacteria may have already started to consume the amino acids present in meat. These changes can lead to the production of alternative catabolites during aging, such as ammonia, amines or organic sulfides, which probably increased the pH values of the control group (Hughes et al. 2014; A. Bekhit et al. 2021).

### 3.2 Instrumental Meat Color

The visual differences of discolored and control meat detected after vacuum‐packaging persisted until the end of shelf‐life. This affirmation was also validated when measuring color for a second time, close to the end of shelf‐life. Discolored meat pieces had significant higher L*, lower a* and non‐significant higher b* values, when compared to the control samples.

However, the evolution of color values during the wet‐aging process varied between control and discolored samples. It was observed that the L* values (lightness) of the discolored samples remained relatively constant throughout wet‐aging. This trend is consistent with findings from other studies, which showed that L* values of vacuum‐packaged *M. Longissimus* remained constant during aging up to 21 days (Hur et al. 2013; Mahmood et al. 2017; Mitacek et al. 2019). In contrast, the control samples exhibited an increase of L* during wet‐aging. This difference in meat lightness can be attributed to changes in meat pH, maybe caused by larger protein degradation and higher water‐holding capacity, which could be responsible for swelling of the fibers and shrinkage in the space between myofibrils (Wu et al. 2020). As pH increases, meat tends to reflect and scatter more light, thereby producing brighter surface appearance (Hanani et al. 2022; Bruce et al. 2004).

Regarding the a* values (redness), both discolored and control samples showed a noticeable decrease of redness during the wet‐aging process, which declined 2.95 and 2.72 points, respectively. These results are aligned with those from an experiment on *M. Longissimus lumborum*, where a* values decreased by 2.2 points after 21 days of aging (Mitacek et al. 2019). Another study found that redness remained constant in *M. Longissimus thoracis* between Day 7 and 21 of wet‐aging (Mahmood et al. 2017), while a separate study showed a large improvement in a* values (approximately 5.4 points) in *M. Longissimus dorsi* after 21 days (Hur et al. 2013).

It was documented that changes in a* values were probably the most sensitive indicator of meat oxidation and even minimal differences in redness (0.95 points) could be detected by trained judges (Juárez et al. 2012; R. R. Mancini et al. 2022). Our results suggested that the larger a* values obtained from the control samples may be beneficial for meat quality, despite these samples having a higher ultimate pH compared to the discolored samples. Conversely, lower redness could adversely affect consumer purchasing decisions, particularly when a* values drop below the critical threshold of 14.5, potentially leading to product rejection (Holman et al. 2017). The average a* scores observed in the discolored samples can potentially pose a substantial challenge in the marketability of these muscles.

In addition, significantly higher *h_a/b_* values in discolored meat at both testing points indicated a greater metmyoglobin superficial accumulation compared to the control. This could be probably due to lower activity of metmyoglobin‐reducing enzymes in discolored beef (Maciel et al. 2021; Farouk and Wieliczko 2003). Higher C* values were also measured from the control samples, which indicated more vivid and saturated colors than discolored beef (Gagaoua et al. 2018). These results reaffirm that control samples had an overall better color profile than the discolored meat pieces, which further highlighted the optical variations between them.

### 3.3 Total Viable Counts

In principle, the muscles of healthy animals are considered sterile, which means that microbial cross‐contamination generally occurs during processing, storage and distribution (Erkmen and Bozoglu 2016). The main source of microbial contamination in beef originated when meat comes in short contact with surfaces that have high bacterial loads, such as hides and/or intestinal material. Other sources of contamination may include the environment of the processing facilities, equipment, and workers (Alexa et al. 2024). For this reason, TVC was evaluated to get a general overview on how bacterial populations interact in meat, after packaging and until the end of shelf‐life. Meat microbiological contamination can result not only in spoilage and rejection from consumers, but can potentially derive in a public health risk, due to the transmission of foodborne pathogens (Alexa et al. 2024). In our experiment, discolored and control meat showed no significant differences in TVC values at any point of the experiment. Overall, contamination of discolored and control samples was relatively low after packaging, probably due to high‐quality standards kept during carcass processing at the slaughterhouse. It has been stated that TVC can be minimized by the implementation of accurate temperature control during the whole production chain, however it cannot completely avoid the growth of psychrophilic microorganisms that can proliferate at low temperatures (Hernández‐Macedo et al. 2011; Colton et al. 2024). During the following wet‐aging, the TVC figures increased at almost the same pace in both groups, reaching approximately log_10_ 6 CFU/g before expiration (Table 2). This suggested that bacteria were able to further utilize the nutrients available in meat for their biological activities at a similar rate. It was published that when TVC exceeded the limit of log_10_ 7 CFU/g, beef is no longer acceptable for human consumption (Zinoviadou et al. 2009). At the end of shelf‐life, both treatments would have been considered safe for consumers, at least from a microbiological point of view. Microbial growth did not appear to be a determining factor in reducing oxygen partial pressure to the critical threshold necessary to initiate metmyoglobin formation. Previous studies have concluded that surface discoloration can be reached before meat can be considered as spoiled (Colton et al. 2024). This conclusion was also supported by the comparable low TVC observed in both discolored and control meat samples (*p* < 0.05), indicating that microbial activity likely had a negligible impact on myoglobin oxidation.

### 3.4 Anaerobic Counts

Anaerobic counts also showed no significant differences between discolored and control samples, both at the beginning and towards the end of the experiment. Nevertheless, it was seen that the anaerobic counts increased at the end of wet‐aging, indicating that these conditions were ideal for their proliferation (Table 2). The absence of oxygen in vacuum‐packaging systems and the release of carbon dioxide by meat tissues probably favored the growth of anaerobic microorganisms during wet aging (Simard et al. 1984). It seemed that anaerobic bacteria were able to employ the metabolites found in discolored and normal meat at a similar rate for their growth and multiplication. This information also suggested that likely comparable substrates were present in both types of meat.

### 3.5 Microbial Identification of Vacuum‐Packaged Beef After Packaging via MALDI‐TOF (MS)

**FIGURE 4:**
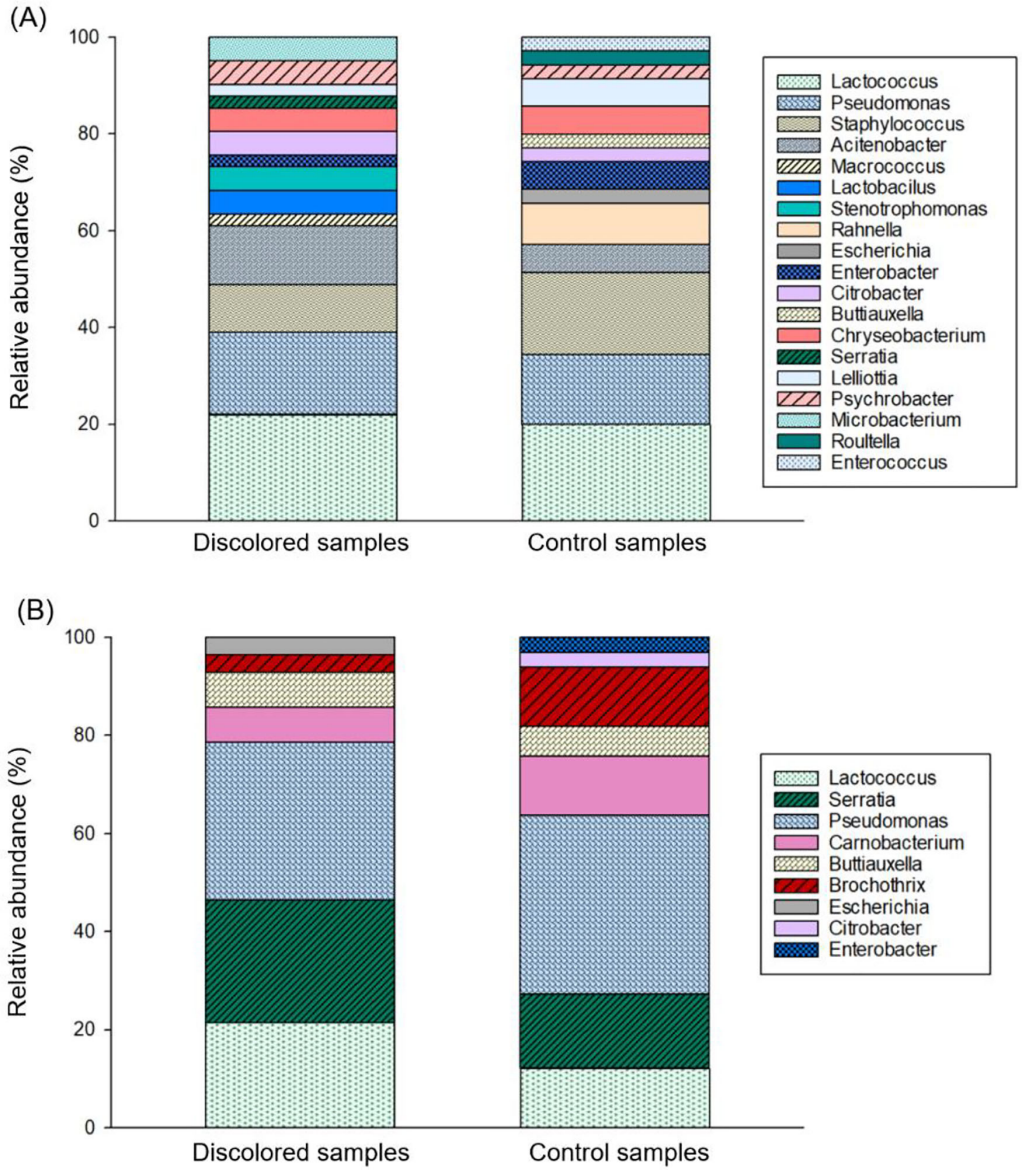
Relative abundance (%) of the bacteria found after packaging (A), and before the end of shelf‐life (B), in discolored and control beef muscles, using MALDI‐TOF MS at a genus level.

The diversity of bacterial populations identified after packaging (Figure 4A) was notably greater than that observed in samples collected toward the end of shelf‐life (Figure 4B **)**. These findings suggested that at the beginning of the wet‐aging process, conditions were likely more conducive to the growth of a wider range of bacterial species, possibly due to higher nutrient availability at this stage. In contrast, a narrower spectrum of bacterial populations was detected as the meat aged under anaerobic conditions, with the harsher vacuum environment limiting the diversity of bacteria that could thrive. In addition, competitive interactions among bacterial groups may have further reduced diversity, as some bacterial species inhibited the growth of others.

Between slaughtering and vacuum‐packaging, there was a window of approximately 96 h, in which aerobic conditions prevailed (Figure 1). Once the environment shifted to an oxygen‐deprived state, the growth of anaerobic bacteria could have been favored for a few days. Due to this reason, by the time that the samples were analyzed at this first inspection point, they already contained an important fraction of LAB, such as *Lactococcus*. It was hypothesized that a higher growth of LAB can have negative repercussions in the development of greenish colors on meat (Hernández‐Macedo et al. 2011). In our case, however, the discolored samples after packaging had in average only 2% more LAB relative abundance than the control, reducing the chances that discoloration problems originated by the superficial growth of this type of bacteria. Nonetheless, it could be anticipated that the primary microbial populations growing at the surface of discolored and control samples after packaging would consist of strict or facultative aerobic bacteria.

The results of microbial identification also showed that discolored‐ and control samples after packaging shared about 80% of their relative microbiome abundance, with 61% and 57% representing the variation of the four most common microbial populations, which consisted of *Lactococcus* (*L. garvieae, L. lactis, L. piscium*, and *L. raffinolactis*), *Pseudomonas* (*P. lundensis, P. antartica, P. taetrolens, P. gessardii, P. fragi, P. libanensis*, and *P. trivialis*), *Staphylococcus* (*S. equorum, S. pasteuri, S. aureus*, and *S. warneri*), and *Acitenobacter* (*A. guillouiae* and *A. johnsonii*). Smaller fractions of less abundant bacteria such as *Enterobacter* (*E. kobei*)*, Citrobacter* (*C. braakii and C. koseri*)*, Chryseobacterium* (*C. vrystaatense*)*, Lelliottia* (*L. amnigena*), and *Psychrobacter* (*P. immobilis*) were also shared for both groups and accounted for around 19.5% and 23% and of the relative abundance of bacteria in discolored and control samples after packaging, respectively.

The main differences in the genera of the microbiome of both groups comprised on the presence of *Macrococcus* (*M. caseolyticus*), *Lactobacillus* (*L. garvieae* and *L. sakei*), *Stenotrophomonas* (*S. acidaminiphila* and *S. rhizophila*), *Serratia* (*S. fonticola*), and *Microbacterium* (*M. maritypicum* and *M. liquefaciens*), that were exclusively found on discolored meat; while small percentages of *Escherichia* (*E. coli*), *Buttiauxella* (*B.gavinieae*), *Raoutella* (*R. ornithinolytica*), and *Enterococcus* (*E. faecalis*) were only found in the control meat. These differences represented around 19.5% and 20% of the relative abundance in discolored and control muscles after packaging, respectively.

### 3.6 Microbiological Identification of Discolored Meat After Packaging via MALDI‐TOF (MS)

Bacteria from the genus *Macrococcus* have been isolated from animal skin, meat, and milk products and from food processing factories, with no relevant consequences for human health (Mazhar et al. 2018; Kloos et al. 1998). *Lactobacillus garvieae* has been identified as a causative agent of mastitis (Gibello et al. 2016), therefore it can be found in milk products and different kinds of meat, including beef. Its importance has been recognized in the clinical field as an opportunistic human pathogen, but no specific correlations have been found between its growth and meat discoloration (Ricci et al. 2013; Ferrario et al. 2014). *Lactobacillus sakei* has been related to spoilage processes and slime formation in cured meat products, with no relevant importance in discoloration of fresh meat (Lorenzo et al. 2018). The growth of bacteria from the genus *Stenotrophomonas* have been mostly associated with the environment, plants and soil and have been recognized to play an important role in nitrogen and sulfur cycles, and probably therefore their high affinity for proteins, but with no specific impact in meat discoloration (Ryan et al. 2009). Members of the *Serratia* family are recognized as Enterobacteriaceae, which can be opportunistic pathogens and that can grow in vacuum‐packaged beef at low temperatures. It has been recognized to produce esters and CO_2_. It has been related with the defect in vacuum‐packaged beef known as blown‐pack, but no correlation has been found with discoloration of meat (Hernández‐Macedo et al. 2011; Fusco et al. 2018; Dorn‐In et al. 2023). Members of the genus *Microbacterium* have been previously identified in dairy products, soil, insects, marine environments and raw meat, and poultry, but no information has been published which associates these bacteria with meat discoloration (Dave and Ghaly 2011; Mounier et al. 2017).

### 3.7 Control Meat After Packaging

Three members of the Enterobacteriaceae family were exclusively found in the control meat samples right after packaging: *E. coli, Buttiauxella*, and *Raoutella*. Of all the three genera of bacteria, *E. coli* is the best known, and some strains could be identified as potential human pathogens such as shigatoxigenic *E. coli*, while other strains are harmless. Enterobacteriaceae can be found in the intestines of the animals, that is why they are usually related to possible cross‐contamination (Nychas et al. 2008; Lorenzo et al. 2018), and to temperature abuse in chilled vacuum‐packaged beef (Iulietto et al. 2015). It was generally assumed that Enterobacteriaceae can cause metabolites with unpleasant odors and greenish discoloration in meat due to their metabolism (Hernández‐Macedo et al. 2011; Colton et al. 2024). Interestingly, because Enterobacteriaceae were found also in discolored meat samples before the end of shelf‐life, no direct relationships with discoloration processes could be established.

*Enterococcus* are a group of bacteria that can also be found in the gastrointestinal tract of animals, which can cause potential contamination with meat (Franz et al. 1999). Due to their robust nature, *Enterococci* can grow in several food media and cause different infections in humans (Fusco et al. 2018). It was published that there was a 17% of probability to find *E. faecalis* in fresh beef samples (Dave and Ghaly 2011). *Enterococcus* species can synthetize H_2_S_2_ as a side product from amino acids breakdown, which can contribute to greenish discoloration in beef (Dave and Ghaly 2011; Iulietto et al. 2015; A. Bekhit et al. 2021). Also in this case, even if a correlation with possible discoloration was found in the literature, it showed no practical effects in this study, because Enterococcus could not be identified in any of the samples after wet‐aging.

### 3.8 Microbial Identification of Vacuum‐Packaged Beef Before Expiration Date

As mentioned before, as storage time increases, there was a notorious reduction in the diversity of the bacterial populations, due to the challenging conditions imparted from oxygen absence. Before the end of shelf‐life, six genera of bacteria dominated around 97% and 94% of the microbiome of discolored and control samples, respectively. As expected, all of these microorganisms were able flourish under anaerobic conditions as strict or facultative anaerobic. In discolored meat, the presence of *E. coli* was minimal, but suggested that probable cross‐contamination originated from bacteria of the intestinal tract of the animals could survive after wet‐aging and can be problematic in terms of food safety. However, its presence was poorly correlated with meat discoloration (A. Bekhit et al. 2021).

On the other hand, two bacterial populations were found exclusively in the control group: *Enterobacter kobei* and *Citrobacter koseri*. Both species have been commonly found in fresh meat and have been catalogued as potential opportunistic pathogens (Nychas et al. 2008). In other words, these bacteria can cause serious infections in vulnerable populations such as infants, young children, pregnant women, and immunosuppressed patients (Fusco et al. 2018).

*Enterobacter* species were related to spoilage processes on modified‐atmosphere‐packaged (MAP) meat (Iulietto et al. 2015). However, no certain interactions with discoloration of vacuum‐packaged meat were found in the literature, showing little or no importance in this topic. It was also published that Staphylococci can be used as an indicator of poor sanitary conditions in the slaughterhouse, as it was mostly related with contact with the skin and/or mucus membranes of the animals (Cuny et al. 2024).

### 3.9 Multivariate Statistical Analysis

**FIGURE 5:**
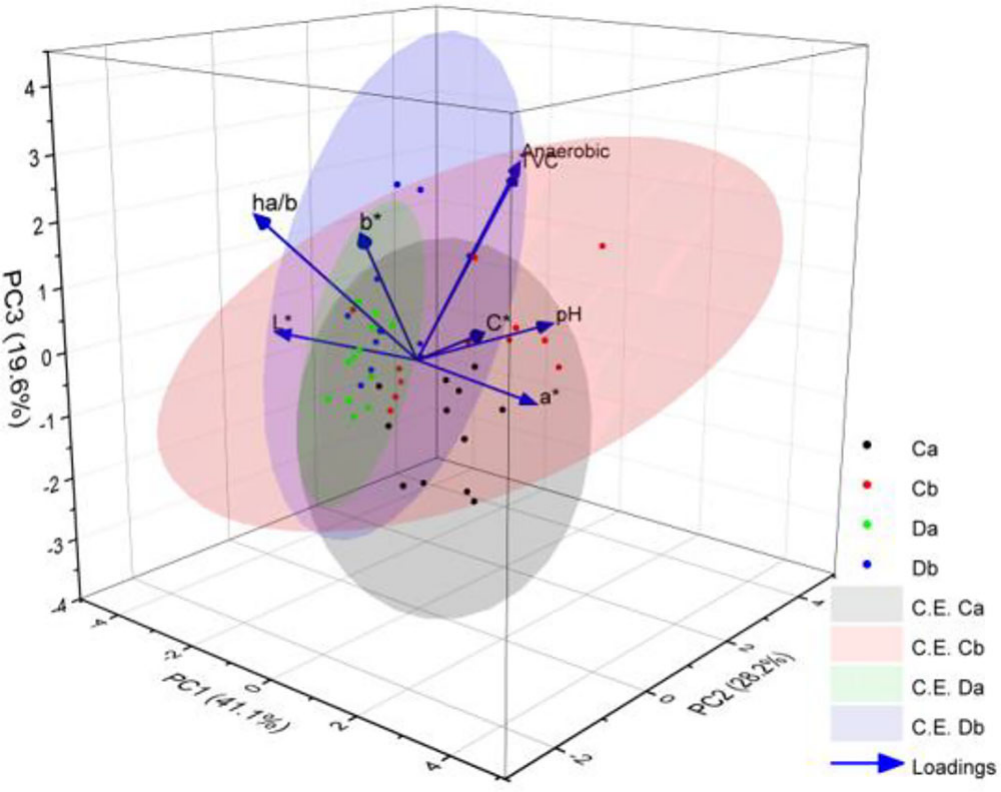
Principal component analysis (PCA) plot from all eight variables studied (loadings). The first three principal components account for 88.9% of the total variance. PCA1 accounts for 41.1%, PC2 for 28.2% and PC3 for 19.6% of the variation. C are the controls and D are discolored samples, a) immediately after packaging and b) just before the expiration date. The location of the variables in the three‐dimensional multivariate space was determined according to their component loadings, which represent the correlations between the variable and the components. The ellipses of the four treatments studied were depicted at a 95% confidence (C.E.).

A PCA was generated from the data matrix containing the eight variables studied in this manuscript. The results indicated that the first three PC explained 88.9% of the total variance (Figure 5 **)**. Among the variables, L*, a*, and C* strongly influenced PC1, whereas TVC, anaerobic counts and *h_a/b_* had a relatively smaller impact on PC2. The PCA plot demonstrated that TVC and anaerobic counts were positively correlated between themselves but inversely correlated with b* and C* scores. This could mean that meat with high bacterial numbers (TVC and anaerobic) tended to create duller and less yellowish colors in beef (because b* coordinates were not negative in any case). It was also shown that some of the values go in the direction of TVC and anaerobic loadings, but without any major influence. It could also be evidenced that when L* and pH values were compared, it suggested that brighter meat color corresponded to lower pH values.

The PCA plot also revealed that individual samples formed four different circles, underscoring differences among the meat groups. Discolored samples after packaging were more tightly clustered, while control samples before expiration date showed a broader dispersion across the multivariate space, reflecting a greater variability in their measured values.

### 3.10 Network Diagram

**FIGURE 6:**
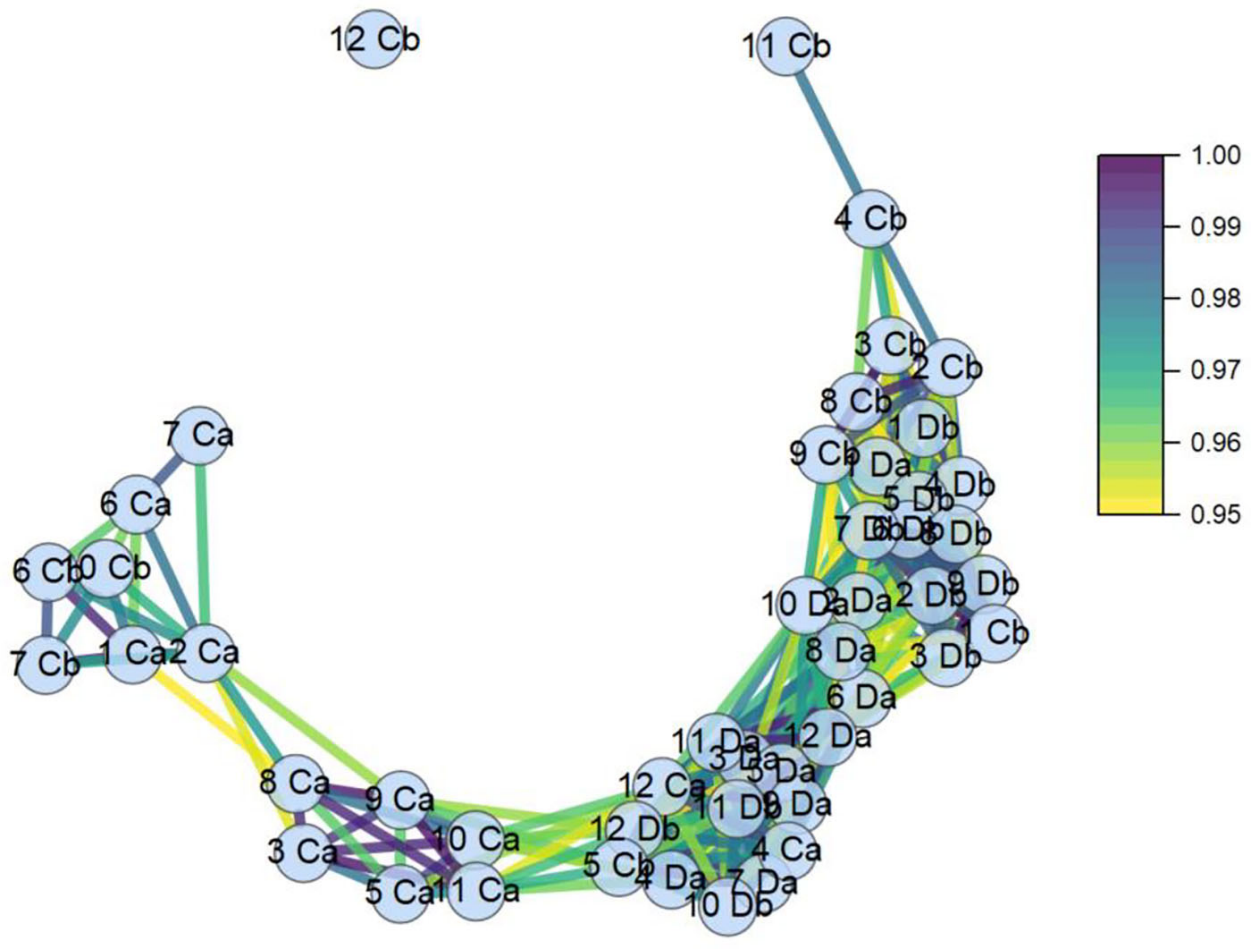
Network diagram showing the associations between all samples involved in this experiment. The nodes are connected if their correlation is higher than 0.95. Also, the color mapping suggested that a higher degree of correlation between the values can be seen from darker lines. For this diagram, samples were categorized as Da (discolored after packaging), Ca (control after packaging), Db (discolored before expiration date), and Cb (control before expiration date).

The primary advantage of this diagram lies in its ability to provide a comprehensive overview of the correlations among the different samples across all measured parameters described in this study. From Figure 6, it can be seen that three dispersed clusters of samples were generated, with only one clear outsider. The analysis of the individual clusters revealed that Da samples exhibited a higher degree of correlation among themselves, as that they tended to accumulate at the middle of the plot. This contrasted with the control samples before expiration date, which were distributed between all the other clusters. Most of the control samples after packaging are condensed at one of the extremes, together with a few control samples before expiration date. This information reaffirmed the results from the PCA and showed that control samples before expiration shared higher similarities with discolored muscles at both testing points. This information confirmed the physicochemical and microbiological differences that existed between discolored and control meat samples when measured at both testing points.

## 4 Conclusion

This study confirms the effectiveness of the MALDI‐TOF (MS) method for identifying bacterial populations in discolored and control samples of vacuum‐packaged beef at two critical stages: after packaging and before the end of the expiration date. However, the color differences found between these two groups of samples could not be completely explained from a microbiological basis. Discoloration of beef muscles is probably triggered by a complex interaction of pre‐ and post‐mortem factors happening during the muscle‐to‐meat conversion, in which oxygen partial pressure and inherent metmyoglobin reducing activity mechanisms play a crucial role. These differences could exacerbate the discoloration in certain hindquarter muscles, eventually making it visible to consumers. To better understand the underlying mechanisms of discoloration in vacuum‐packaged beef, future research should incorporate robust traceability systems capable of interconnecting the influence of intrinsic and extrinsic factors throughout the production chain.

## Author Contributions

**Alejandro Poveda**: conceptualization, investigation, writing – original draft, methodology, validation, visualization, formal analysis. **Johannes Krell**: visualization, writing – review and editing, methodology. **Volker Heinz**: conceptualization, project administration, funding acquisition, resources. **Nino Terjung**: conceptualization, investigation, funding acquisition, writing – review and editing, methodology, project administration, supervision, data curation. **Igor Tomasevic**: conceptualization, investigation, writing – review and editing, methodology, supervision. **Monika Gibis**: conceptualization, investigation, funding acquisition, writing – review and editing, supervision, methodology, resources, project administration.

## Conflicts of Interest

The authors declare no conflicts of interest.
